# Metabolomics of Cerebrospinal Fluid in Multiple Sclerosis Compared With Healthy Controls: A Pilot Study

**DOI:** 10.3389/fneur.2022.874121

**Published:** 2022-05-26

**Authors:** Michal Židó, David Kačer, Karel Valeš, Zuzana Svobodová, Denisa Zimová, Ivana Štětkárová

**Affiliations:** ^1^Department of Neurology, Third Faculty of Medicine, Charles University, Prague, Czechia; ^2^Department of Neurology, Faculty Hospital Královské Vinohrady, Prague, Czechia; ^3^National Institute of Mental Health, Klecany, Czechia; ^4^Institute of Physiology, Academy of Sciences of the Czech Republic (ASCR), Prague, Czechia

**Keywords:** multiple sclerosis, CSF, metabolomics, biomarker, fatty acids, amino acids, arginine

## Abstract

**Background:**

Multiple sclerosis (MS) is a chronic autoimmune disease of the central nervous system (CNS) leading to the loss of myelin and axons. Diagnosis is based on clinical findings, MRI, and analysis of cerebrospinal fluid (CSF). CSF is an ultrafiltrate of plasma and reflects inflammatory processes in the CNS. The aim of this study was to perform metabolomics analysis of CSF in patients after the first attack of MS and healthy controls and try to find new specific analytes for MS including those potentially predicting disease activities at the onset.

**Methods:**

We collected CSF from 19 patients (16 females, aged 19–55 years) after the first attack of clinical symptoms who fulfilled revised McDonald criteria of MS and CSF of 19 controls (16 females, aged 19–50 years). Analyses of CSF samples were provided using the high-performance liquid chromatography system coupled with a mass spectrometer with a high-resolution detector (TripleTOF 5600, AB Sciex, Canada).

**Results:**

Approximately 130 selected analytes were identified, and 30 of them were verified. During the targeted analysis, a significant decrease in arginine and histidine and a less significant decrease in the levels of asparagine, leucine/isoleucine, and tryptophan, together with a significant increase of palmitic acid in the patient group, were found.

**Conclusion:**

We observed significant differences in amino and fatty acids in the CSF of newly diagnosed patients with MS in comparison with controls. The most significant changes were observed in levels of arginine, histidine, and palmitic acid that may predict inflammatory disease activity. Further studies are necessary to support these findings as potential biomarkers of MS.

## Introduction

The increasing incidence of autoimmune diseases in the population poses a serious problem to contemporary medicine. One such condition is multiple sclerosis (MS), a severe autoimmune inflammatory disease of the central nervous system (CNS) that primarily affects the young, working age population. The disease is chronic and progressive in nature and leads to a disability due to multifocal demyelination and axonal loss. The CNS white matter is predominantly involved (1).

The exact cause of MS is still unknown. It is believed that various pathogenic mechanisms may play a role in disease development, including genetic and environmental factors. The pathology of MS is characterized by demyelination of the axons in the CNS as a result of malfunctioning autoimmune processes. Initially, the myelin loss is reversed by the oligodendrocytes. However, repetitive loss and repair of the myelin, microglial activation, and leukocyte infiltration due to increased permeability of the blood-brain barrier (BBB) along with decreased oligodendrocyte efficiency result in long-term axonal degeneration, neuronal death, and plaque formation. The subsequent accumulation of neurological damage and progression of neurodegenerative processes ultimately lead to irreversible neurological disability (2). Diagnosis of MS is based on a combination of clinical (3) and magnetic resonance imaging (MRI) observations, supported by findings in cerebrospinal fluid (CSF) (oligoclonal bands or increased intrathecal immunoglobulin production) (4).

Cerebrospinal fluid analysis remains a valuable, supporting diagnostic test for MS (5). It reflects the inflammatory processes occurring in the CNS in detail. Metabolomics is the systematic study of unique chemical fingerprints that specific cellular processes leave behind. It targets metabolites—small molecular substrates and products of metabolism. This metabolic profiling can help to gain insight into the current state of cellular metabolism.

The desire for early treatment of MS and to assign each patient to the most suitable therapy is hampered by the lack of useful prognostic biomarkers that could predict disease progression, severity, and responses to treatment. In recent years, several studies have investigated the metabolomics of CSF in MS. The reported results suggested significant differences between patients with MS and healthy controls. The significant changes were found in amino acid and lipid groups (6–11), where they reported decreased levels of tyrosine, leucine, and phenylalanine. Other studies (12, 13) found significant differences in CSF fatty acids between the groups. One of the most notable findings was an increase in glutamate levels in patients with MS with active lesions observed in CSF (14, 15) and in serum (16, 17). Several contradicting reports on myoinositol and choline results have been published. Some authors (9, 18, 19) found increased levels of choline and myoinositol, whereas others have observed a decreased value (20).

The aim of this study was to analyze the metabolomics of CSF in patients with MS and compare them with healthy controls and establish a statistically significant list of differences in CSF of patients with MS and potentially identify MS-specific analytes. We have performed an untargeted approach, but based on our results, we focused on the groups of amino acids and fatty acids, as they play important roles in the pathophysiology of MS. Fatty acids form a part of myelin, and amino acids have a close connection to immunological processes building elements of proteins and functioning as neurotransmitters.

## Materials and Methods

### Recruitment of Patients

Recruitment of suspected patients with MS was conducted using the database of the Centre for Multiple Sclerosis, Third Faculty of Medicine, Charles University and University Hospital Královské Vinohrady (FNKV). We have already selected 19 patients (16 females, 3 males, mean age of 36 years) after the first attack of clinical signs and symptoms, who fulfilled revised McDonald criteria for MS from 2017 (5) (onset of the disease, without specific Disease Modifying Drugs (DMD) treatment, with MS-specific MRI finding, and meet CSF criteria for diagnosis of MS) and 19 controls (16 females 3 males, mean age of 35 years) with normal neurological status, normal CSF findings, and no anamnesis of autoimmune disorders. Subjects chosen to be part of the control group mostly suffer from sensory disturbances, dizziness, polymorphic complaints, or headache but with negative objective clinical or paraclinical findings to define a specific neurological disease. This group was defined as symptomatic controls [according to already published data (21)] aged between 18 and 55 years, with no use of psychopharmacological drugs (which can alter CSF composition) and no history of other autoimmune disorders.

All subjects participating in this study provided consent and received a full explanation about the entire study. All subjects underwent baseline serum and CSF assessment and clinical and brain and/or spinal cord MRI examination according to their clinical signs and symptoms. In the case of the patient group with MS, the CSF was on average collected on the 15th day from the beginning of clinical signs and symptoms; two exceptions (patients no. 12 and 18) had their CSF collected 4 months from the onset of symptoms, and a further two patients (no. 11 and 19) saw their symptoms last for years before CSF collection.

### CSF Sample Preparation

Cerebrospinal fluid samples collected by lumbar puncture were carefully thawed on ice. After thawing, the samples for the first analysis (untargeted metabolomics) were vortexed, and 100 μl was transferred into a precooled Eppendorf tube. An immediate addition of 400 μl of ice-cold ACN:MeOH mixture (1:1, v/v) was used to maintain the MeOH:ACN:H_2_O (2:2:1, v/v) ratio. The samples were vortexed for 30 s and consequently incubated for 1 h at −20°C. Incubation was followed by 10 min of centrifugation at 13,000 rpm at 4°C for 20 min. The supernatant was transferred and evaporated to dryness by speedVAc. The dry extract was reconstituted in 100 μl of H_2_O:MeOH (1:1, v/v) and sonicated for 10 min. The insoluble debris was removed by centrifugation (13,000 rpm for 10 min at 4°C), and the supernatant was transferred into a vial and directly analyzed by liquid chromatography–mass spectrometry (LC–MS).

Samples for targeted analysis of amino acids were prepared the same way as is described above. Samples for analysis of fatty acids were extracted according to the modified Bligh and Dyer method (Bligh and Dyer, 1959). Notably, 100 μl of thawed CSF was mixed with 400 μl of chloroform-methanol 50:50 (v/v) with added internal standard and placed in an ultrasound bath for 5 min. Extraction was performed for 2 h at 4°C, followed by the addition of 100 μl of mili-Q water, 15 s of vortexing, and centrifugation at 4,000 rpm for 10 min at 4°C. The upper and lower phases divided by thin protein discs were pooled together and dried under a nitrogen stream. Samples were reconstituted with 100 μl of MeOH/IPA/H_2_O 65:35:5 (v/v/v), vortexed for 10 s, and sonicated for 5 min before injection.

### High-Performance Liquid Chromatography With Tandem Mass Spectrometry (HPLC-MS/MS) Analysis

All analyses were performed on a Thermo Ultimate 3000 coupled with a high-resolution AB Sciex TripleTOF 5600 mass spectrometer. During the first round of analyses, untargeted metabolomics analyses were all the collected samples, together with quality control samples and blank samples, injected in both positive and negative (ESI+, ESI–) modes using the information-dependent acquisition (IDA) method. Samples were analyzed *via* separation on a Phenomenex high-performance liquid chromatography (HPLC) Kinetex C18 2.6 μm 150 × 3 mm column. The column temperature was maintained constant at 30°C. The mobile phase was composed of A = 0.1% formic acid in water and B = 0.1% formic acid in 100% MeOH for both positive and negative modes, the linear elution gradient from 5% B (0–2 min) to 100% B (18–23 min) was applied, the initial gradient conditions were restored within 2 min (23–25 min), and the last 5 min of the HPLC method (25–30 min) were applied to maintain the beginning conditions. The flow rates were 220 μl min^−1^, and the sample injection volume was 5 μl. Samples were held in an autosampler at 4°C, and each sample was injected twice for each mass/charge (*m*/*z*) range (50–500 Da, 500–1,200 Da). The ESI source conditions were set as follows: ion source gas 1 (GS1) 35 psi, ion source gas 2 (GS2) 30 psi, curtain gas (CUR) 25 psi, ion spray voltage 4,000 V, and source temperature 300°C.

The method that targeted the determination of small metabolites including amino acids was slightly modified from the previous one. The separation was achieved on the Phenomenex Kinetex C18 2.6 μm 150 × 3 mm HPLC column with the following eluent system: A = 0.1% HCOOH, 2.5 mM NFPA in water, and B = 0.1% HCOOH in MeOH. A linear gradient (5–100% B) from 2nd to 12th min was used, with a flow rate of 0.22 ml min^−1^. The injection volume was set to 5 μl. Samples were held in an autosampler at 4°C, and each sample was injected twice for an *m*/*z* range of 50–500 Da. The ESI source conditions were set as follows: ion GS1 35 psi, ion GS2 30 psi, CUR 25 psi, ion spray voltage 4,500 V, and source temperature 400°C.

Separation of targeted fatty acids (palmitic, stearic, oleic, and arachidonic) was performed with C18 reverse-phase column – Phenomenex Kinetex C18, 2.6 μm, 150 × 3 mm column at 45°C. Mobile phase A consisted of 1% 1 M NH_4_Ac and 0.1% acetic acid in water and mobile phase B consisted of acetonitrile/isopropanol 7:3 (v/v) with 1% 1 M NH_4_Ac and 0.1% acetic acid, with an injection volume of 5 μl. The following gradient was applied: 1 min − 50% of B; 3 min – linear gradient from 50% B to 80% B; 8 min – linear gradient from 80% B to 90% B; 13 min – linear gradient from 90% B to 100% B; 15 min − 100% B; 17 min − 50% B; 20 min − 50% B; with a constant flow rate of the mobile phase of 300 μl/min. Data were acquired in TOF MS full scan and IDA in both ESI+ and ESI- modes. The source parameters were set as follows: GAS1: 50 psi; GAS2: 45 psi; CUR: 30 psi; TEM: 300°C; ISVF: 5,500 V in positive mode and −4,500 V in negative mode, respectively.

### Data Processing

The liquid chromatography with tandem mass spectrometry (LC-MS/MS) data was processed using Sciex OS software (version 1.3 with Formula Finder plug-in, AB SCIEX, Canada), which offers the evaluation of the retention time (RT) and *m*/*z* variability of the experiment. MarkerView software (version 1.3.1, AB SCIEX, Canada) was used in the second step to process raw LC-HRMS data (peak detection, alignment, data filtering, and determining the m/z ratio, RT, and ion peak area for each sample). Data mining was performed by the program algorithm—the peak intensity cutoff was set at 100 cps. Peak settings were achieved using RT and *m*/*z* tolerances of 0.1 min and 0.005 Da, respectively. Monoisotopic peaks alone were considered to reduce mass redundancy and enhance the selection of a true molecular feature. Finally, mass signals differentially expressed by the control and case study samples (Sclerosis Multiplex) were identified by applying an additional filtering procedure with fold change (<1.5) and *t*-test (*p* > 0.05). This whole procedure is necessary for the elimination of the background and contaminants and preserved the true biological mass signals from the LC-HRMS data. The following steps were carried out using the MetaboAnalyst 5.0 Web Server. Acquired and filtered data from MetaboAnalyst 5.0 were, in the following step, verified with previously acquired data from targeted analyses (analyses of ~80 standards include amino acids, fatty acids, and other small metabolites already set into the spectral library using Sciex OS software).

### Statistical Data Analysis

The Student's *t*-test was used for the comparison between the control group and sclerosis multiplex group, followed by the application of the Benjamini-Hochberg false discovery rate (FDR) correction for multiple comparisons to minimize false positives.

## Results

The demographic and clinical data of the suspected patients with MS are summarized in Supplementary Table S1. All recruited patients with MS had hyperintensive lesions on MRI that meet revised McDonald criteria (5). All the patients with MS presented brain MRI lesions in the supratentorial region, and 11 of them also had hyperintensive lesions in the spinal cord MRI. All suspected patients with MS had positive IgG oligoclonal bands (OCB) in CSF with a median of 8 IgG OCB in the whole spectrum (ranging from 1 to 17), negative aquaporin 4 antibodies, and negative myelin oligodendrocyte glycoprotein antibodies in serum and CSF. More detailed information about the CSF findings and OCB is presented in Supplementary Table S2. Patients with MS and control group subjects have the normal level of proteins in CSF. Some of the patients with MS (no. 1, 3, 6, 9, 10, 12, and 16) presented increased numbers of mononuclear cells in CSF, but without any signs of neuroinfection or other kinds of neuroinflammation other than that caused by MS.

Subjects from both groups (suspected patients with MS and control group) had no history of psychopharmacological drug use. Four patients with MS (no. 6, 11, 14, and 18) had a history of hormonal anticonception use, and another three patients with MS (no. 6, 11, and 12) had a history of using levothyroxinum at a daily dose of 50 μg.

**Figure 1:**
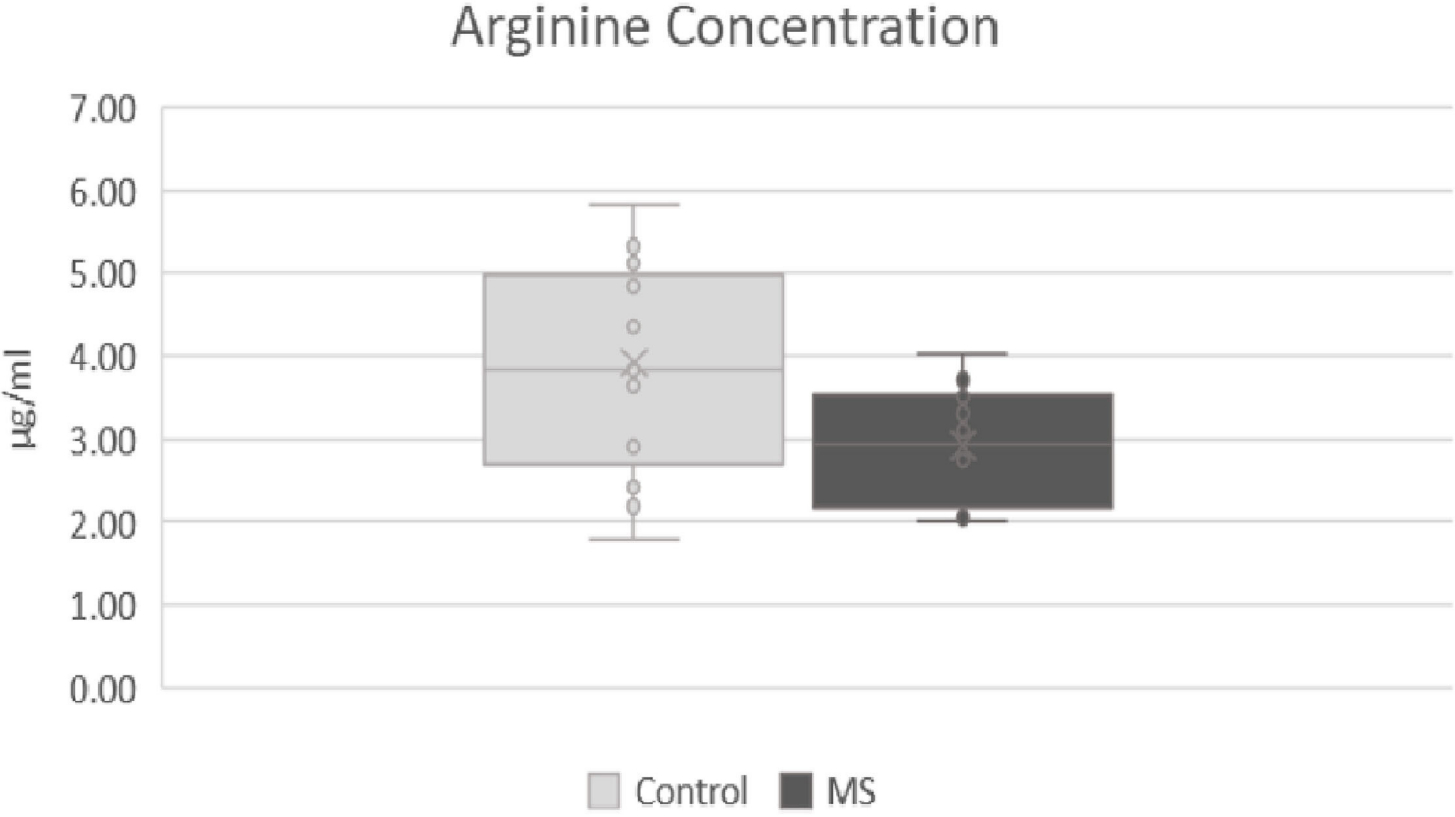
Comparison of levels of arginine in patients after first attack of clinical symptoms fulfilling revised McDonald criteria and control group. MS, patients after first attack of clinical symptoms fulfilling revised McDonald criteria; control, control group.

**Figure 2:**
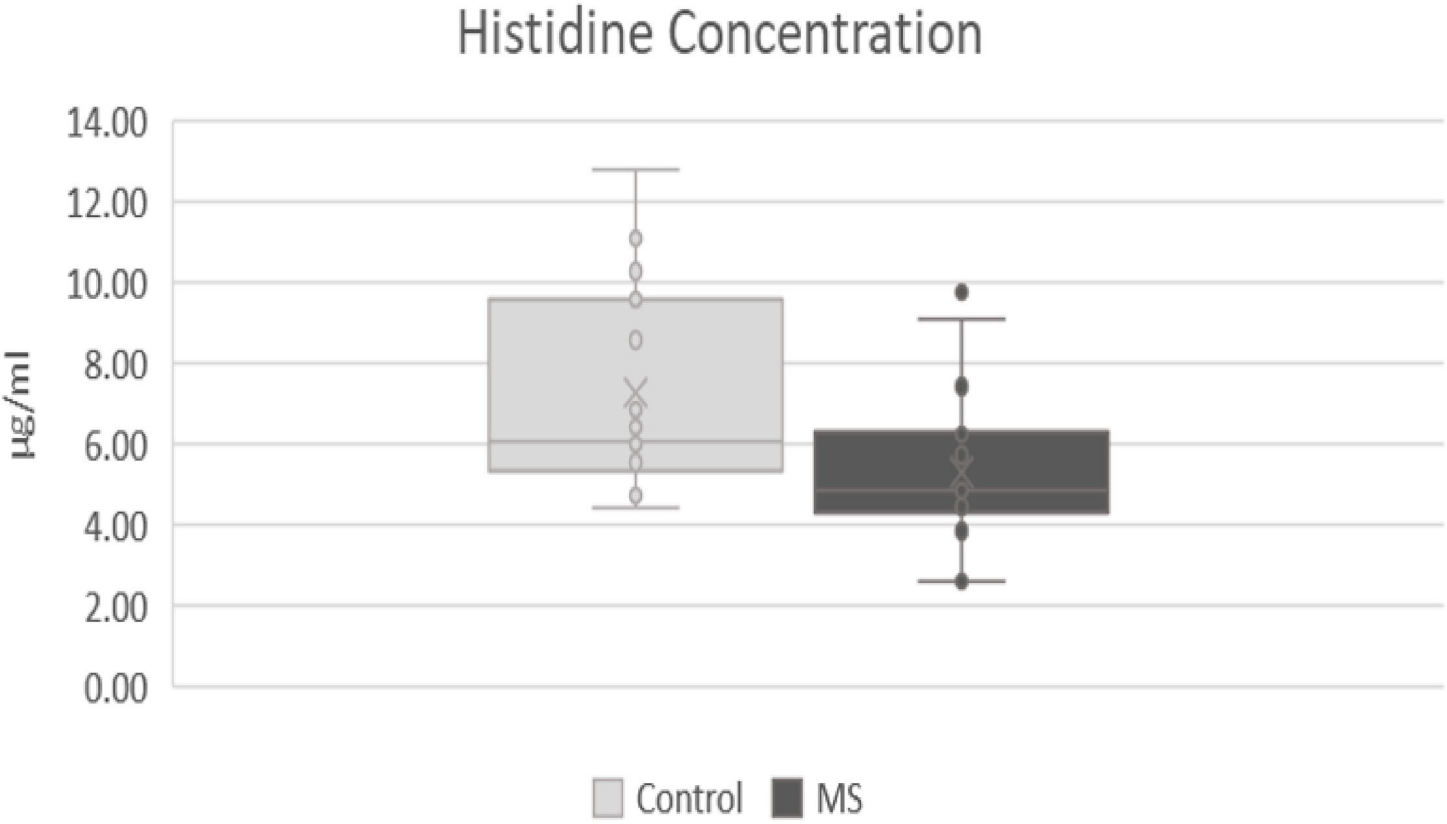
Comparison of levels of histidine in patients after first attack of clinical symptoms fulfilling revised McDonald criteria and control group. MS, patients after first attack of clinical symptoms fulfilling revised McDonald criteria; control, control group.

**Figure 3:**
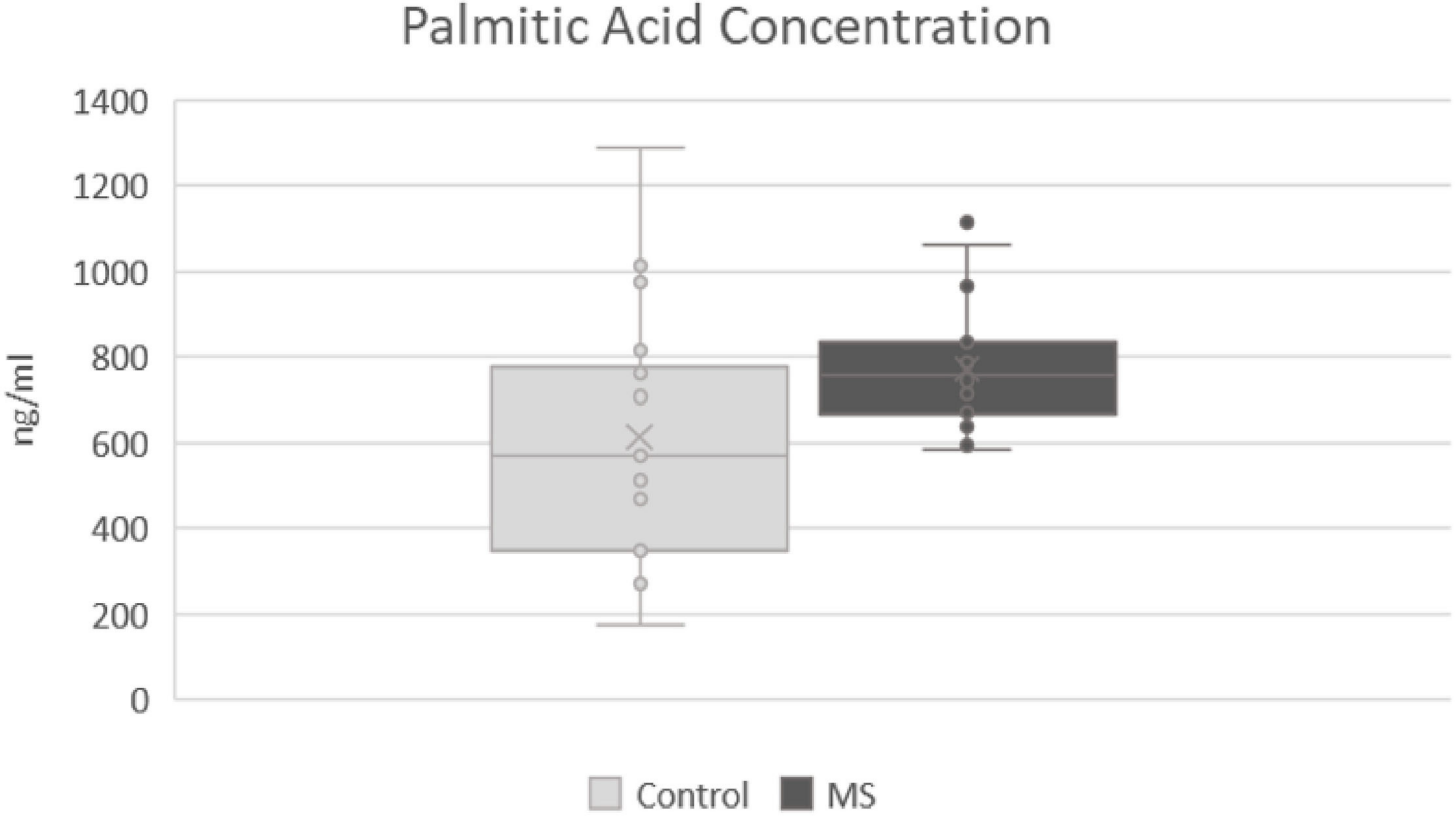
Comparison of levels of palmitic acid in patients after first attack of clinical symptoms fulfilling revised McDonald criteria and control group. MS, patients after first attack of clinical symptoms fulfilling revised McDonald criteria; control, control group.

In the metabolic pathways of fatty acids and amino acids, significant differences were found in the CSF between the two mentioned groups. The most significant differences were observed in arginine, histidine, and palmitic acid. Statistically, the most significant results were found in the level of arginine (*p*-value: 0.007), where a lower level of arginine was observed in patients with MS (mean responses 2.91909) and then in the control group (mean responses 3.91752) (Figure 1). In histidine (*p*-value: 0.012), we found a significantly lower level in patients with MS (mean responses 5.31349) than in the control group (mean responses 7.27157) (Figure 2). The level of palmitic acid (*p*-value: 0.039) was significantly higher in patients with MS (mean responses 1.28959) than in the control group (mean responses 1.02343) (Figure 3). In contrast, no statistically significant changes were observed in asparagine (*p*-value: 0.1135), leucine/isoleucine (*p*-value: 0.1325), and tryptophan (*p*-value: 0.1384), whereby the levels of these analytes were lower in the patients with MS than in the control group, respectively (for more details, refer to Table 1).

**Table 1:** Detailed results of cerebrospinal fluid (CSF) analysis.

**Peak name**	**m/z**	***t*-value**	***p*-value**	**Mean of response in MSp**	**Mean of response in controls**	**Standard deviation in MSp**	**Standard deviation in controls**
Arginine	175.1194	−2.11990	0.00704	2.91909916	3.9175231	0.6900299	1.2255753
Histidine	156.0772	−2.07235	0.01201	5.31349736	7.2715724	2.0317250	2.5291640
Palmitic acid	257.2494	1.745883	0.03968	1.28959022	1.0234377	0.2596009	0.4869935
Asparagine	133.0797	−1.62355	0.11358	11.5585353	14.179993	4.1724773	5.8151468
Leucine/isoleucine	132.1017	−1.55221	0.13252	0.04613981	0.1116484	0.0757918	0.1719756
Tryptophane	205.0977	−1.51469	0.13841	0.79384388	1.1638709	0.7753553	0.7488412
Lysine	147.113	−1.36629	01806	5.45858649	6.4206882	1.8475467	2.5147676
Cystein	122.043	−1.36132	0.18933	0.0014778	0.1835504	0.0064416	0.5980968
Threonine	120.0662	1.315061	0.19673	0.40598326	0.2515651	0.3813894	0.3502126
Phenylalanine	166.086	−1.32739	0.19894	0.00881132	0.0509445	0.0273785	0.1391445
Glutamate	148.0604	1.193139	0.24041	0.263029	0.294388	0.209961	0.241989
Oleic acid	283.2638	0.868702	0.39148	0.47934379	0.243562	0.9783302	0.6825356
Arachidonic acid	305.2454	−0.94153	0.35309	0.35530687	0.523647	0.4516842	0.6516032
Stearic acid	285.2803	−0.1646	0.87019	0.21131172	0.2398059	0.4726169	0.6035298

*MSp, patients after first attack of clinical symptoms fulfilling revised McDonald criteria; m/z, mass spectrum*.

The quality control (QC) sample represents an analytical approach employing a sample produced and utilized by the operators to guarantee the quality of the measured data and results. Due to the number of collected and analyzed samples (including QC samples), the analyses required a runtime of ~120 h. Random injections of QC samples throughout the long runtime ensured no signal changes while performing the experiment. The analytical system provided uniform profiles and yielded excellent reproducibility even after the entire analytical runtime.

## Discussion

In this study, we showed statistically significant differences in the CSF metabolomics of patients after the first attack of clinical signs of symptoms fulfilling revised McDonald criteria for MS in comparison to healthy controls. The most significant differences were found in the groups of amino acids and fatty acids, especially with decreased levels of arginine, histidine, asparagine, leucine/isoleucine, and tryptophan and an increased level of palmitic acid. These CSF metabolomics results could become new potential markers in the early stage of MS and can potentially be used in the prediction of the disease severity in the future. In the future, the specificity of these potentially MS-specific analytes needs to be verified.

Cerebrospinal fluid is the most important biological sample that can help us to understand the pathology of MS. CSF can be used for measurements of various soluble markers and cell populations. It is also considered the “gold standard” matrix in MS diagnostics. However, CSF collection is an invasive procedure and is, therefore, only collected on rare occasions. The majority of proteins found in CSF are blood-derived. These proteins cross the BBB and reach the CSF compartment *via* passive diffusion. CSF is a better medium to identify potential biomarkers of MS due to the lower amount of different proteins. It reflects the actual state of CNS through possible inflammatory processes.

Several authors have already published the results of CSF metabolomics in patients with MS (e.g., 5, 6, 9, and 10); however, they collected samples from patients with MS in various stages of the disease with different DMD treatments and specific pharmacological history. In our study, we have clearly homogenous MS and control patient groups. In the case of MS, we focused on patients after their first attack of clinical signs and symptoms, without a history of psychopharmacological drug use and fulfilling revised McDonald criteria for MS, without any kind of specific medication such as DMDs or high-dosage corticosteroid pulse. Some other authors recruited people in the control group from different types of neurological diseases, even inflammatory CNS diseases such as meningoencephalitis. We used symptomatic controls (21), meaning patients with non-specific complaints, suffering mostly from sensory disturbances, dizziness, or headache, with negative objective clinical or paraclinical findings.

Few reports from last year even focused on the metabolomics of blood samples of patients with MS (10, 22) and found decreased levels in amino acids, more specifically phenylalanine, tyrosine, and tryptophan (10) and modified asparagine and carnitine (22). In our study, we focused solely on CSF; therefore, we cannot relevantly compare our findings.

Amino acids play an important role in the CNS and in the immune system, not only as a “building material” for proteins but also as precursors of neurotransmitters, which have important roles in inflammatory processes and pathogenesis of MS (23). Some authors have published a decreased level of arginine in patients with MS (6–9). This observation can be explained by its mechanism in the metabolism of nitrite oxide (NO). L-arginine is a precursor of NO, which is a neurotransmitter with a potential role in the pathogenesis of MS (24). NO is synthesized from arginine in the regular way by endothelial NO synthase (eNOS) and neuronal NO synthase (nNOS). These forms produce low concentrations of NO in a calcium-dependent way. At sites of inflammation, acute/active lesions in MS, another form of enzyme starts to produce NO. This form of enzyme produces high concentrations of NO and is not dependent on calcium concentrations (25, 26). In MS, the concentrations of NO were found to be increased, especially in locations of active lesions (24, 27). In this study, we did not study NO; however, we found a significantly lower level of arginine in our patients with MS after their first clinical attack. We can speculate that arginine can be a suitable analyte of disease activity at the early onset of MS, but we cannot confirm its specificity for MS. In the next steps, it should be compared with other types of inflammations in the CNS.

Histidine, which was also found to be decreased in MS (6–9), is a precursor of neurotransmitter histamine, synthesized in histaminergic neurons of the tuberomammillary nucleus in the posterior third of the hypothalamus. Histamine plays an important role in the inflammatory processes as well as in the pathogenesis of MS, but its role is not yet certainly established (28). Several studies found increased levels of histamine in the CSF of patients with MS (29–31), which can potentially explain the lower levels of histidine, its precursor. In our study, we have found decreased levels of histidine in patients with MS; therefore, our results are in agreement with others.

In already published reports, leucine/isoleucine and branched-chained amino acid have been found to be decreased in MS (6–9). Leucine/isoleucine has an important role in protein synthesis, as a key nitrogen donor, and in cell growth and proliferation. During inflammation, there is an increased synthesis of many different proteins and increased immune cell growth, which means increased demand for branched-chained amino acids resulting in a decrease of leucine/isoleucine. In our study, we found a slightly decreased level of leucine/isoleucine, but not a statistically significant one.

Some authors have been studying glutamate and found its high level in the CSF of patients with MS (14, 15). In this study, we did not find significant differences in glutamate in MS compared with the control group. One explanation of this result could be that we have investigated subjects in the early stage of MS, where the acute inflammatory process of demyelination is supposed to be at the beginning of the disease. Other authors have studied patients in different stages of MS, meaning that the most dominant process of axonal destruction followed by an increase in extracellular glutamate may occur in the later phase of MS (32).

We also observed a significantly increased level of palmitic acid that was in agreement with several studies (13, 33). During an acute attack of MS, there is a loss of myelin, consisting mostly of lipids. Myelin plays an important role in normal nerve transmission. In demyelination, an increase in fatty acids as the basic components of lipids and myelin (34) can be found.

Contrary to our results, some authors have reported a decreased level of palmitic acid in MS (12, 13), probably due to the heterogeneity of patients with MS with early and/or late stages of the disease with remyelination or demyelination. The decrease of palmitic acid might be explained by the process of remyelination, in which myelin is being recreated by oligodendrocytes and thereby consuming fatty acids as basic components of myelin (34). In this study, we recruited patients with MS in the early stage of disease with an ongoing first attack of the disease, meaning that the process of demyelination might be the dominant mechanism and, therefore, with increased levels of palmitic acid.

## Conclusion

In this study, we concluded that arginine, histidine, and palmitic acid may be used as analytes potentially specific for the early stages of MS. Their specificity need yet to be verified, but they still may be used in verification of ongoing inflammation or active lesion in CNS in the early stages of MS.

Decreased levels of arginine and histidine can be explained by their role as precursors of neurotransmitters (arginine as a precursor of NO and histidine as a precursor of histamine), which are significantly increased in the inflammatory processes of MS, and therefore, precursor consumption is increased.

Palmitic acids, as the basic component of fatty acids, are involved in the demyelination process, and therefore, their increased levels may be found in the early stages of MS.

The potential use and specificity of these analytes (arginine, histidine, and palmitic acid) need to be more thoroughly examined in a larger group of patients with MS to establish their role in disease progression and compare them to other inflammatory diseases of CNS.

## Data Availability Statement

The raw data supporting the conclusions of this article will be made available by the authors, without undue reservation.

## Ethics Statement

The studies involving human participants were reviewed and approved by Ethics Committee of University Hospital Kralovske Vinohrady under number EK-VP/64/0/20. The patients/participants provided their written informed consent to participate in this study.

## Author Contributions

MŽ contributed to manuscript writing, collecting and analyzing the data. IŠ was a major contributor in manuscript writing and final approval. MŽ, DK, and KV analyzed and interpreted the patient data. MŽ, ZS, and DZ performed clinical investigation and data collection. All authors contributed to the article and approved the submitted version.

## Funding

This research was supported by the Charles University Research Program Cooperatio 38, Neurosciences Charles University, GAUK 120121 and SVVV 260533/SVV/2022. Funding sources had no involvement in the study design; collection, analysis, and interpretation of data; in the writing of the report; or in the decision to submit the manuscript for publication.

## Conflict of Interest

The authors declare that the research was conducted in the absence of any commercial or financial relationships that could be construed as a potential conflict of interest.

## Publisher's Note

All claims expressed in this article are solely those of the authors and do not necessarily represent those of their affiliated organizations, or those of the publisher, the editors and the reviewers. Any product that may be evaluated in this article, or claim that may be made by its manufacturer, is not guaranteed or endorsed by the publisher.
