# Supplementary material for: Metabolomics of Cerebrospinal Fluid in Multiple Sclerosis Compared With Healthy Controls: A Pilot Study
Source: Front Neurol. 2022 May 26;13:874121. doi: 10.3389/fneur.2022.874121 (PMC9178205; doi:10.3389/fneur.2022.874121)
Supplement: Supplementary file 1 [file Data_Sheet_1.PDF]

Supplementary materials

Table s1: Clinical data of patients after the first attack of clinical symptoms fulfilling revised McDonald criteria

Identification number	Sex	Age	Medical history	Chronic medication	Clinical findings and symptoms	EDSS**
**1**	M	38	0	0	Central scotoma of LE, retrobulbar pain in LE	1.5
**2**	F	26	0	0	Weakness of L leg	2.5
**3**	F	36	Atopic eczema	0	Vision impairment on LE	2.5
**4**	F	27	0	0	Acroparest. of legs	2.5
**5**	F	43	Appendectomy, cholecystectomy	Hormonal anticonception	Parest. of L arm	2
**6**	F	32	Hypothyr.	Levothyroxinum 50 mcg daily	0	2.5
**7**	M	28	0	0	Dysest. of R extremities	1
**8**	F	27	WPW sy.	0	NS vertigo, parest. of R and L arm	1
**9**	F	33	NS sight impairment	0	Parest. of upper extremities	1.5
**10**	F	18	Asthma bronchiale, depression*	Hormonal anticonception	Migrating parest.	2.5
**11**	F	40	Hyperthyr.	Levothyroxinum 50 mcg daily	Paroxysmal dysest. provoked by heat	1.5
**12**	F	54	AH, obesity, hypothyr.	Levothyroxinum 137 mg daily, omeprazolum 20 mg daily, hydrochlorothiazidum 25 mg daily, allopurinolum 300 mg daily	NS vertigo, headache, parest. of L arm	2
**13**	F	31	0	Hormonal anticonception	Blurred vision on LE	1.5
**14**	M	43	0	0	Retrobulbar pain bilat., blurred vision on LE, parest.	1
**15**	F	33	0	0	Quadruparest.	1.5
**16**	F	25	0	0	Parest. of L arm	2
**17**	F	25	0	Hormonal anticonception	Headache, diplopia	1.5
**18**	F	40	0	0	Acroparest. of upper extremities, migrating dysest.	0
**19**	F	49	Appendectomy	0	Parest. of L arm	2.5

Notes: M = male; F = female; AH = arterial hypertension; NS = non-specific; hyperthyr. = hyperthyreosis; hypothyr. = hypothyreosis; WPW = Wolf-Parkinson-White; sy. = syndrome; LE = left eye; RE = right eye; L = left; R = right; parest. = paresthesia; dysest. = dysesthesia; EDSS = expanded disability status scale

* in this patient depression was not treated by any specific antidepressants

** EDSS counting was done by certified neurologist DZ

Table s2: CSF results of patients after the first attack of clinical symptoms fulfilling revised McDonald criteria

Identification number	IEF of IgG in CSF	IEF of IgA in CSF	IEF of IgM in CSF	IEF of kappa in CSF	IEF of lambda in CSF	P in CSF (g/l)	Glc in CSF (mmol/l)	Mononucl. in CSF (µl)
**1**	5alk	3alk	4alk	2alk	1alk	0.31	5.5	19
**2**	7alk	10alk	9alk	8alk	5alk	0.32	3.85	4
**3**	7alk	11alk	21alk	10ac	2alk	0.26	3.31	40
**4**	9alk	9alk	9alk	8ac	7alk	0.25	3.12	12
**5**	1alk	1alk	1alk	0	0	0.27	3.1	2
**6**	13alk	11alk	9alk	15ac	12ac	0.26	3.44	30
**7**	17 whole grad.	11alk	22alk	16ac	11ac	0.32	3.41	4
**8**	3paraneu	10alk	8alk	10ac	8ac	0.15	3.35	8
**9**	2paraneu	2alk	0	6paraneu	1paraneu	0.43	3.31	27
**10**	10alk	7alk	5ac	9ac	10alk	0.45	2.94	42
**11**	14alk	12alk	5alk	12alk	0	0.25	3.36	2
**12**	6ac	8alk	8alk	8alk	0	0.43	3.11	34
**13**	9alk	8alk	8alk	7alk	8alk	0.41	3.04	13
**14**	16 whole grad.	14 whole grad.	13alk	16ac	4alk	0.4	3.72	22
**15**	15alk	15lak	8ac	6ac	0	0.33	2.75	5
**16**	12 whole grad.	4paraneu	7ac	2ac	0	0.2	3.27	30
**17**	10paraneu	9paraneu	4ac	4paraneu	6ac 2paraneu	0.19	3.85	2
**18**	9 whole grad.	14alk	14alk	11alk	10alk	0.37	3.21	2
**19**	2alk	1ac	3alk	3lak	6alk	0.21	3.47	3

Notes: CSF = cerebrospinal fluid; IEF = isoelectric focusing; P = protein; Glc = glucose; Mononucl. = mononuclear cells; alk = alkaline; whole grad. = whole gradient; paraneu = paraneutral; ac = acidic
